# Oxytocin Signaling in Mouse Taste Buds

**DOI:** 10.1371/journal.pone.0011980

**Published:** 2010-08-05

**Authors:** Michael S. Sinclair, Isabel Perea-Martinez, Gennady Dvoryanchikov, Masahide Yoshida, Katsuhiko Nishimori, Stephen D. Roper, Nirupa Chaudhari

**Affiliations:** 1 Program in Neurosciences, University of Miami Miller School of Medicine, Miami, Florida, United States of America; 2 Department of Physiology & Biophysics, University of Miami Miller School of Medicine, Miami, Florida, United States of America; 3 Department of Molecular and Cell Biology, Graduate School of Agricultural Science, Tohoku University, Sendai, Miyagi, Japan; Duke University, United States of America

## Abstract

**Background:**

The neuropeptide, oxytocin (OXT), acts on brain circuits to inhibit food intake. Mutant mice lacking OXT (OXT knockout) overconsume salty and sweet (i.e. sucrose, saccharin) solutions. We asked if OXT might also act on taste buds via its receptor, OXTR.

**Methodology/Principal Findings:**

Using RT-PCR, we detected the expression of OXTR in taste buds throughout the oral cavity, but not in adjacent non-taste lingual epithelium. By immunostaining tissues from OXTR-YFP knock-in mice, we found that OXTR is expressed in a subset of Glial-like (Type I) taste cells, and also in cells on the periphery of taste buds. Single-cell RT-PCR confirmed this cell-type assignment. Using Ca^2+^ imaging, we observed that physiologically appropriate concentrations of OXT evoked [Ca^2+^]_i_ mobilization in a subset of taste cells (EC_50_ ∼33 nM). OXT-evoked responses were significantly inhibited by the OXTR antagonist, L-371,257. Isolated OXT-responsive taste cells were neither Receptor (Type II) nor Presynaptic (Type III) cells, consistent with our immunofluorescence observations. We also investigated the source of OXT peptide that may act on taste cells. Both RT-PCR and immunostaining suggest that the OXT peptide is not produced in taste buds or in their associated nerves. Finally, we also examined the morphology of taste buds from mice that lack OXTR. Taste buds and their constituent cell types appeared very similar in mice with two, one or no copies of the OXTR gene.

**Conclusions/Significance:**

We conclude that OXT elicits Ca^2+^ signals via OXTR in murine taste buds. OXT-responsive cells are most likely a subset of Glial-like (Type I) taste cells. OXT itself is not produced locally in taste tissue and is likely delivered through the circulation. Loss of OXTR does not grossly alter the morphology of any of the cell types contained in taste buds. Instead, we speculate that OXT-responsive Glial-like (Type I) taste bud cells modulate taste signaling and afferent sensory output. Such modulation would complement central pathways of appetite regulation that employ circulating homeostatic and satiety signals.

## Introduction

Oxytocin (OXT), a nonapeptide hormone classically known to facilitate lactation and parturition, is also a central neuropeptide that influences a host of social and other behaviors [1]. The peripheral actions of OXT are elicited principally following its release into the bloodstream from hypothalamic magnocellular neurons with terminals in the pituitary. The central effects of OXT are in response to release from magnocellular dendrites and axonal projections of parvocellular neurons [2].

Several lines of evidence link OXT with feeding behaviors in rodents and humans. Oxytocin is released in areas of the brainstem and hypothalamus involved in appetite regulation [3], [4]. Injections of OXT into the cerebral ventricles of rodents inhibit food and fluid intake [5]–[7]. Relative to wild-type mice, OXT knockout (OXT^−/−^) mice overconsume solutions of saccharin and carbohydrates including sucrose [8]–[10]. Curiously, OXT^−/−^ mice have a normal appetite for palatable, energy-rich lipid emulsions [11]. Thus, in these studies, OXT regulated the intake of sweet, but not all calorie-rich solutions. Additional evidence continues to accumulate linking OXT to appetite and feeding behaviors in rodents [12]. OXT is also associated with appetite regulation in normal and in pathological contexts in humans. For example, *circulating* levels of oxytocin are inversely related to those of the orexigenic peptide, ghrelin [13]. Patients with Prader-Willi syndrome have a reduced number of hypothalamic oxytocinergic neurons, overeat insatiably, and are obese from early childhood [14]. OXT can also modulate salt intake during dehydration, hypovolemia, and/or hypernatremia (reviewed, [15]). OXT^−/−^ mice overconsume NaCl solutions after fluid deprivation when compared to wild-type mice [16]. In summary, OXT influences feeding, but the evidence suggests that some taste qualities are more subject to this influence than others. This raises the possibility that, in addition to the known targets within central circuits, the peripheral taste system may also be a target of OXT signaling.

Hence, we investigated the presence of OXTR in taste buds and asked if OXT can act directly on them to elicit physiological responses. Using RT-PCR as well as knock-in transgenic mice, we found OXTR expressed in a functionally distinct subset of taste cells with glial-like properties. In these cells, physiological concentrations of OXT elicit Ca^2+^ responses via OXTRs. We also asked if the OXT peptide is produced locally in taste tissue. Neither RT-PCR nor immunostaining in taste tissue revealed the presence of OXT in either taste buds or innervating nerve fibers. OXTRs in taste buds thus likely respond to OXT released into circulation from the pituitary. It is possible, then, that peripheral taste organs may be an important additional substrate for the regulation of ingestion by OXT.

## Results

### Taste buds selectively express OXTR

**Figure 1:**
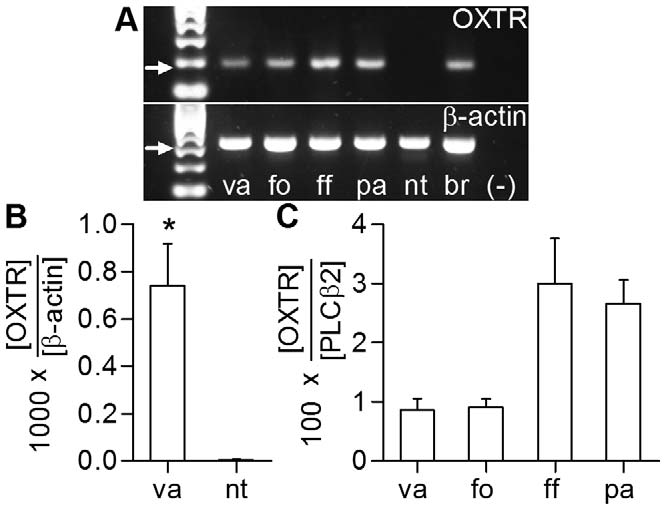
Taste buds selectively express Oxytocin Receptor (OXTR). **A**. RT-PCR for OXTR (upper) and β-actin (lower) on taste epithelium from four areas (va, vallate; fo, foliate; ff, fungiform; pa, palate), non-taste lingual epithelium (nt), brain (br) and water (−). Arrows mark predicted bands (OXTR, 187 bp; β-actin, 328 bp). **B**. Real-time RT-PCR performed in parallel on cDNA from vallate and nontaste epithelia (OXTR mRNA copies normalized to 1000 copies β-actin mRNA) suggests that OXTR expression is taste-selective (* = p<0.05 vallate compared to non-taste). **C**. Real-time RT-PCR on four fields of taste epithelium. OXTR mRNA copy number is normalized to 100 copies of PLCβ2 mRNA. Anterior epithelium (ff) and palate have more copies of OXTR mRNA per unit of taste cell mass compared to posterior taste epithelium (va, fo). Bars depict mean and s.e.m. (n = 3).

To examine whether oxytocin receptor (OXTR) is expressed in taste buds, we performed end-point and real-time RT-PCR on mouse taste epithelia. We found evidence for OXTR mRNA in anterior and posterior taste epithelia that contained taste buds but not in epithelial samples that lacked taste buds (Fig. 1A, B). We also measured the relative expression levels of OXTR using qRT-PCR on taste buds isolated from vallate, foliate, and fungiform papillae, and the palate. When normalized to PLCβ2, a taste-selective phospholipase, we found that expression of OXTR is higher in anterior taste buds (fungiform, palate) compared to posterior (vallate, foliate) taste buds (Fig. 1C). Fungiform and palatal taste buds are innervated by cranial nerve VII (facial), vallate and foliate taste buds by cranial nerve IX (glossopharyngeal). We noted that copy numbers for OXTR mRNA are 30–100-fold lower than for PLCβ2 mRNA. This suggests that OXTR mRNA is expressed at low levels in many cells or at higher levels in only a small number of taste cells. The standard for comparison, PLCβ2, is expressed in 10–30 cells per taste bud [17], [18].

**Figure 2:**
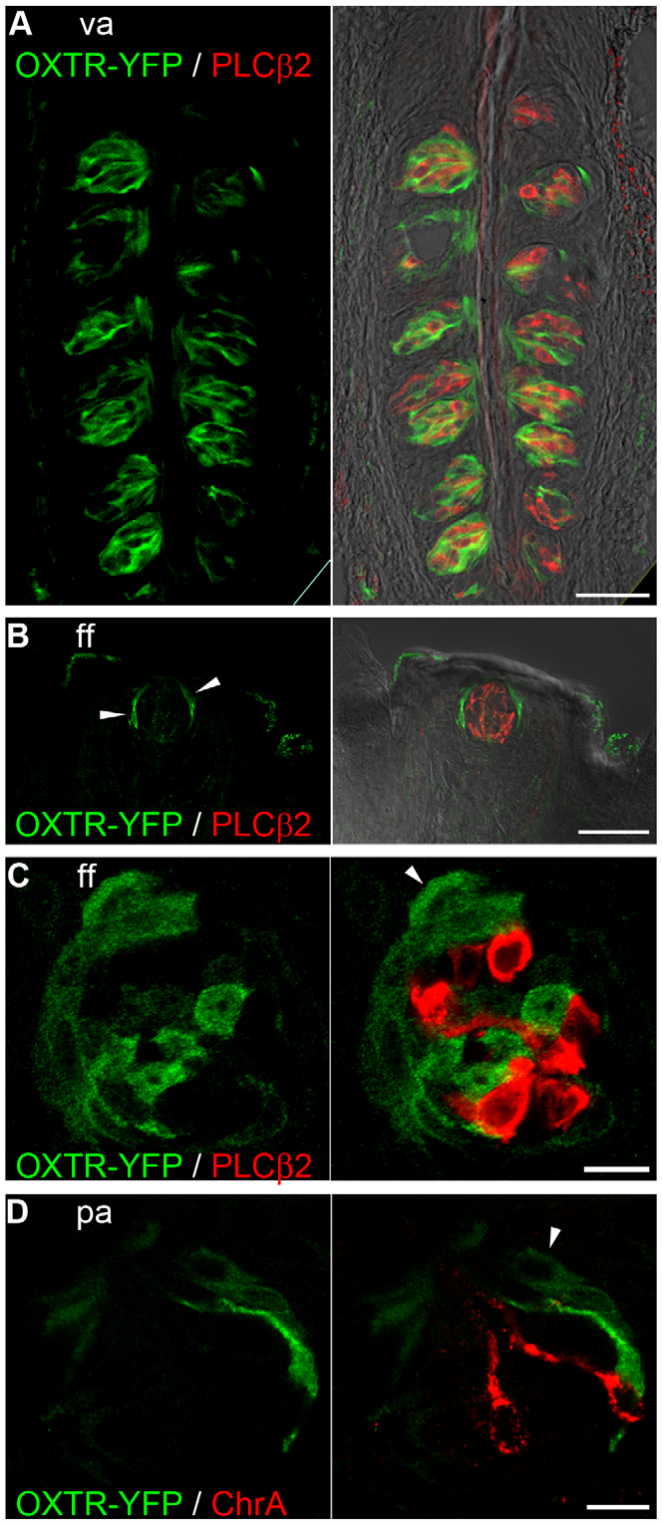
Oxytocin Receptor, is expressed in taste buds but not in Receptor or Presynaptic cells. Cryosections of vallate (va, **A**) or fungiform (ff, **B**) papilla from OXTR-YFP mice, were immunostained for PLCβ2 (red, right) to reveal Receptor cells and taste buds, and with anti-GFP (green) to detect YFP expression. Expression of YFP is limited to taste buds. No YFP was detected in either non-sensory epithelium or underlying connective tissue (revealed by differential interference contrast microscopy in overlay at right). Higher magnification *cross*-sections of fungiform (ff, **C**) or palatal (pa, **D**) taste buds, from OXTR-YFP mice, immunostained for PLCβ2 (**C**) or Chromogranin A (**D**) (red) and YFP (green). OXTR is expressed in cells that are distinct from PLCβ2-positive (Receptor) cells and Chromogranin A-positive (Presynaptic) cells. In many instances, strong GFP fluorescence was detected in cells on the periphery of the taste bud (arrowheads in A–D). Scale bars are 50 µm (A,B) or 10 µm (C,D).

We next asked if OXTR protein could be detected in taste buds, using a fluorescent reporter in transgenic mice. In OXTR-YFP knock-in mice, the coding sequence of one allele of OXTR is replaced with that of the Venus variant of YFP [19]. Consequently, YFP fluorescence is present only in cells that natively express OXTR. Cryosections of taste tissues from lingual and palatal areas displayed prominent fluorescence within all taste buds (Fig. 2A–D) and faint fluorescence in a few nerve fibers under the palatal epithelium (Figure S1). In contrast, YFP could not be detected in surrounding non-taste epithelial cells or in underlying tissues (muscle, salivary glands etc.) (Fig. 2A,B). The expression of YFP as a surrogate for OXTR is consistent with our RT-PCR observations (above), namely that expression in lingual tissue is limited to taste buds.

### OXTR is found in a subset of Glial-like/Type I cells

Mammalian taste buds include three distinct classes of cells [20]–[22] These classes express different complements of genes related to their functions: Receptor (Type II) cells express G-protein coupled taste receptors and transduction components. These cells respond to sweet, bitter and umami taste stimuli by elevating cytoplasmic Ca^2+^ [18], [23]. In contrast, Presynaptic (Type III) cells express neuronal proteins including those associated with synapses and display Ca^2+^ responses that integrate the signals from Receptor cells. Presynaptic cells also respond directly to sour stimuli [18], [23]–[26]. A third class of taste cells (Type I) exhibits glial properties, including mechanisms for clearing neurotransmitters and extracellular K^+^ in the taste bud [27], [28]. Because these classes of cells have markedly different functions, we asked whether OXTR expression is restricted to any one of the classes. We have previously validated PLCβ2 and Chromogranin A as effective markers for Receptor and Presynaptic cells, respectively [18], [29]. In taste buds of OXTR-YFP mice, YFP expression did not overlap with immunostaining for either PLCβ2 (Fig. 2A–C) or Chromogranin A (Fig. 2D). This was the case in four taste fields (vallate, foliate, fungiform, palate). Hence, OXTR-expressing taste bud cells appeared to be neither Receptor cells nor Presynaptic cells. By exclusion, this suggested that OXTR-expressing cells may belong to the less well-described group, the Glial-like (Type I) cells.

**Figure 3:**
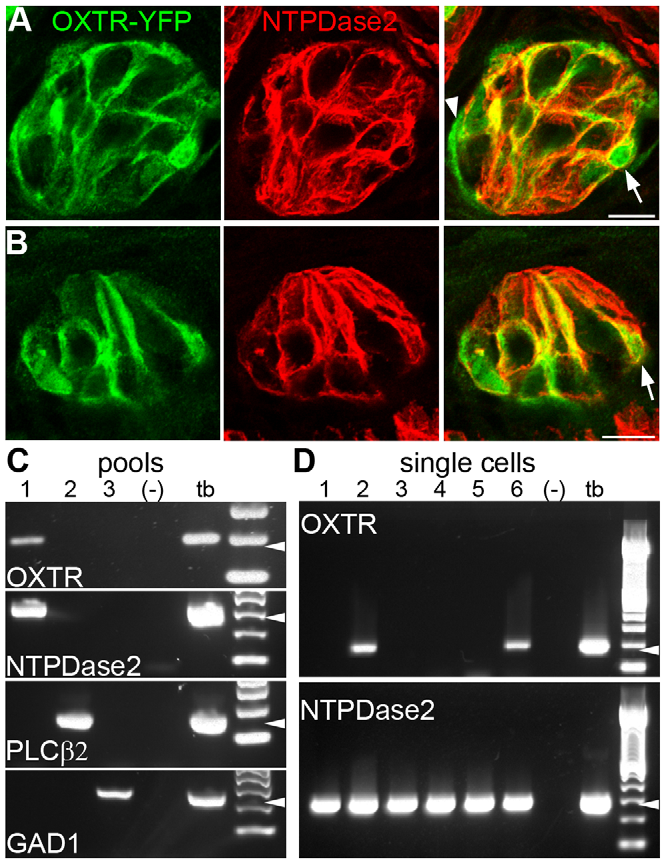
Oxytocin Receptor is present in Glial-like cells in taste buds. **A, B**. Cryosections of vallate papilla from OXTR-YFP mice, cut horizontally (**A**) or vertically (**B**), were immunostained for NTPDase2 (red), a marker for taste glial-like cells, and for YFP (green). YFP is co-expressed in some NTPDase2-positive' cells. Membrane-localized NTPDase2 is detected in YFP-expressing cells (arrows in overlay images, right). YFP is also in cells, particularly at the periphery of the taste bud, that are not associated with NTPDase2 and thus cannot be termed Glial-like (arrowhead). Scale bars are 10 µm. **C**. Pools, each containing 20 taste cells of an identified type, were assayed for OXTR by RT-PCR. Receptor and Presynaptic cells were identified and harvested as GFP-positive cells from taste buds of PLCβ2-GFP (pool #2) or GAD1-GFP (pool #3) mice. To isolate glial-like cells, GFP-negative cells from PLCβ2-GFP x GAD1-GFP double transgenic mice were collected for pool #1. All pools were subjected to RT-PCR for all three diagnostic markers, NTPDase2, PLCβ2, and GAD1 to confirm their identification. OXTR was detected only in the pool (#1) of NTPDase2-expressing, GFP-negative cells. **D**. A typical RT-PCR experiment on 6 individual Glial-like (NTPDase2-positive) cells. OXTR is detected in 2 of the 6 cells. Arrowheads indicate expected product size in each case. Controls are water (−) or whole taste buds (tb).

Next, we immunostained OXTR-YFP taste tissue for Nucleoside Triphosphate Diphosphohydrolase-2 (NTPDase2), a marker for Glial-like (Type I) cells of taste buds [28]. We observed YFP in a subset of cells that expressed NTPDase2 (Fig. 3A,B). Demonstrating co-expression is complicated by the fact that the extracellular epitope of NTPDase2 is localized to the plasma membrane, while YFP is intracellular and soluble. In many cases, YFP-filled cytoplasm was bounded by NTPDase2 on the associated membrane (arrows, Fig. 3A–B). Most YFP-expressing cells had thin processes and irregularly shaped cell bodies (Figure S2) that are morphologically dissimilar to those of Receptor and Presynaptic cells, but similar to those of Glial-like (Type I) cells [20], [28], [30]. We also noted YFP-positive cells on the periphery of taste buds. Peripheral YFP-positive cells typically did not express either NTPDase2 or other markers of differentiated taste cells (arrowheads in Fig. 2B–D; Fig. 3A). OXTR therefore appears to be expressed in a sub-population of Glial-like (Type I) taste cells as well as in cells located on the periphery of taste buds.

To independently verify the cell-type that expresses OXTR, we used RT-PCR on isolated taste cells. First, we assayed RNA from three pools, each containing 20 cells of a single type (see *Methods*). OXTR was not found in either the pool of Receptor (i.e. PLCβ2-expressing) or Presynaptic (i.e. SNAP25-expressing) cells (Fig. 3C, pools 2 and 3). In contrast, the pool containing Glial-like (i.e. NTPDase2-expressing) taste cells was positive for OXTR (Fig. 3C, pool 1). We then conducted single cell RT-PCR on cDNA from individual Glial-like cells (Fig. 3D). In total, 6 out of 29 such individually tested NTPDase2-positive cells were also positive for OXTR. In contrast, none of the individually analyzed 11 Receptor and 18 Presynaptic cells expressed OXTR. The distribution of OXTR only in Glial-like (Type I) cells (rather than in Receptor or Presynaptic cells) is statistically significant (*p<0.05, Chi-square test).

In summary, immunofluorescence in OXTR-YFP tissue and single-cell RT-PCR are consistent in showing that OXTR is expressed in neither Receptor nor Presynaptic taste cells, but instead, is found in a subset of Glial-like taste cells.

### OXT elicits calcium responses from taste cells

**Figure 4:**
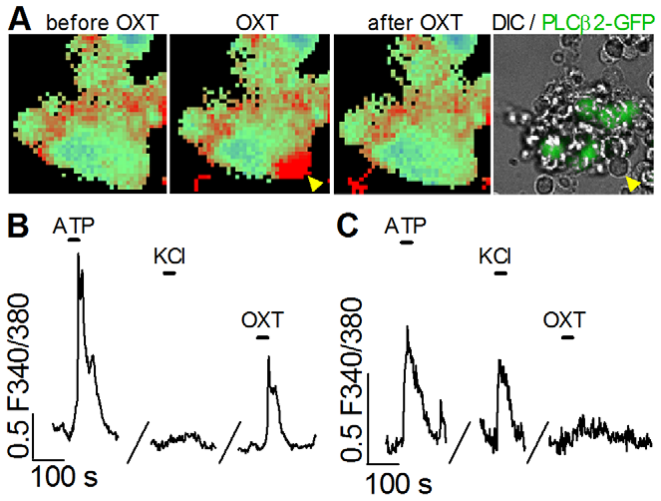
OXT evokes Ca^2+^ responses in taste cells. **A**. Pseudocolored image of fura-2 fluorescence from an isolated taste bud from a PLCβ2-GFP mouse before, during, and after Ca^2+^ mobilization evoked by 30 nM OXT. A cell on the periphery (arrowhead) displayed a Ca^2+^ response (red) that could be replicated with repeated applications of OXT. Image at right shows GFP fluorescence (green) and brightfield micrograph of the same field, overlaid. The OXT-responsive cell is GFP-negative. That is, it is not a Receptor cell. **B**. Ca^2+^ responses (F340/F380 ratio) from a taste cell similar to the one indicated in A. This cell responded robustly to 1 µM ATP but not to depolarization with 50 mM KCl (i.e. it is not a Presynaptic cell). This cell repeatedly displayed Ca^2+^ responses when stimulated with 30 nM OXT. **C**. A Presynaptic cell in a taste bud responded to ATP (1 µM) and to depolarization with 50 mM KCl, but not to 30 nM OXT.

Next, we asked if taste cells are able to respond to physiological concentrations of oxytocin (OXT). In other tissues, activating OXTR elicits Ca^2+^ mobilization from intracellular stores by stimulating phospholipase C to generate inositol trisphosphate [31]. We isolated taste buds and cells from lingual vallate epithelium of PLCβ2-GFP mice, loaded them with the Ca^2+^-sensitive dye Fura-2, and functionally imaged for changes of cellular [Ca^2+^]. When OXT was bath-applied, we observed a small number of taste cells responding with an elevation of intracellular Ca^2+^ (Fig. 4A–B). In many cases, individual OXT-responding cells could easily be resolved, either by virtue of being completely isolated, or being close to the surface of a taste bud (Fig. 4A, arrowhead). Such cells were never GFP-positive when the taste bud preparation was derived from either PLCβ2-GFP or GAD1-GFP mice. Thus, OXT-responsive cells were neither Receptor nor Presynaptic cells.

**Figure 5:**
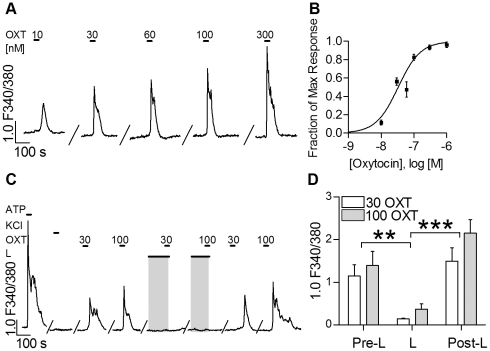
OXT-evoked responses in taste cells are dose-dependent and can be blocked with an OXTR antagonist. **A**. Trace showing OXT responses to increasing doses of OXT (10 nM to 300 nM, for 30 sec each, indicated by bars above traces). **B**. Concentration-response curve, based on peak responses, from 4 separate experiments. Estimated EC_50_ = 33 nM. N = 13 for each point except at 60 nM (N = 5) and 1 µM (N = 8). **C**. An example trace from an OXT-responsive cell, exposed sequentially for 30 sec each to 30 and 100 nM OXT before, during (shaded) and after incubating the preparation in 500 nM L-371,257 (L), an OXTR antagonist. Treatment with L-371,257 reversibly abolished OXT responses at both concentrations of OXT. **D**. Aggregate data (mean +/− s.e.m.) from 3 independent experiments show that L-371,257 significantly inhibits OXT responses in taste cells and that responses to OXT recover after washout of the inhibitor (N = 6; 2-way ANOVA with repeated measures followed by Newman-Keuls post-hoc test. ** = p<0.01 comparing Pre-L to L for each concentration of OXT; *** = p<0.001 comparing Post-L to L for each concentration of OXT). An interval of 20 min with constant flow perfusion of Tyrode buffer elapsed between successive OXT applications.

We examined the responses of taste cells to increasing concentrations of OXT from 10 nM to 1 µM (Fig. 5A) and fit a concentration-response curve to the data (Fig. 5B). We estimated an EC_50_ of 33 nM and saturation at ∼1 µM OXT. These values are similar to those reported for uterine smooth muscle, another peripheral target of OXT [32].

In these imaging experiments, responses evoked by ATP (1–10 µM) served as a test for the viability of isolated taste buds and cells [33], [34]. All OXT-responsive cells also responded to ATP (Fig. 4B, Fig. 5C). Further, we also stimulated the preparation with 50 mM KCl. Presynaptic taste cells respond to depolarization with an influx of Ca^2+^, mediated by voltage-gated Ca channels [18], [25]. OXT-responsive cells never responded to KCl-induced depolarization, although every preparation included Presynaptic cells (i.e. cells that responded to KCl; Fig. 4B,C; Fig. 5C). In summary, the lack of GFP expression and the absence of KCl-evoked Ca^2+^ responses in OXT-sensitive cells is consistent with our single-cell RT-PCR and immunofluorescence data showing that OXTR is not found in Receptor or Presynaptic cells.

### OXT responses in taste cells are blocked by an OXTR antagonist

To ascertain that OXT-evoked responses were mediated by activation of specific receptors, we tested the effect of L-371,257, an OXTR-selective antagonist [35]. L-371,257 (500 nM) significantly decreased responses to OXT (30 nM, 100 nM) (Fig. 5 C,D). OXT responses completely recovered after the antagonist was washed out (Fig. 5C–D). Thus, Ca^2+^ responses to OXT appear to be elicited via the OXTR that we detected by RT-PCR and immunofluorescence (Figs. 1,2,3).

### Oxytocin is not produced locally in taste tissue

**Figure 6:**
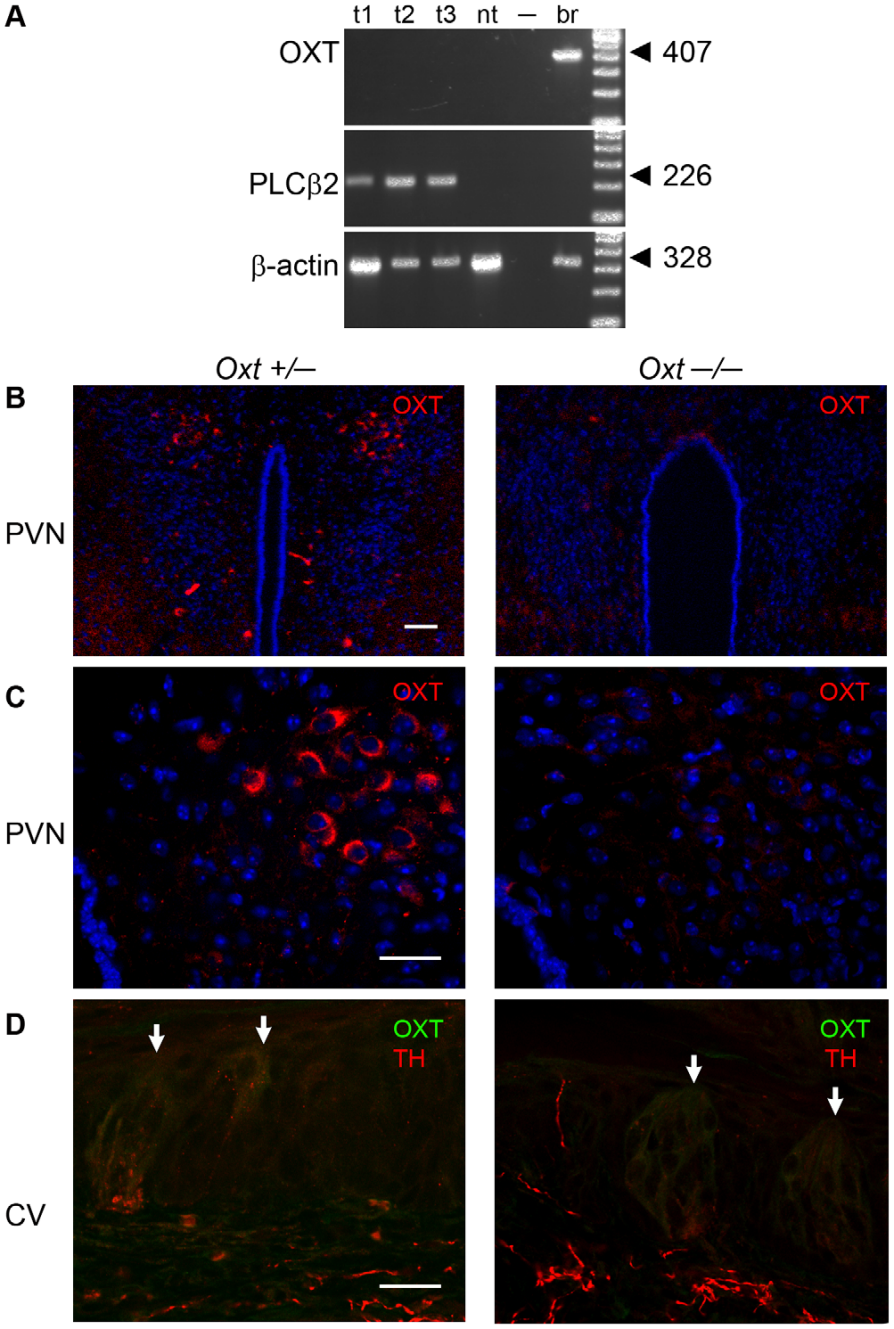
Oxytocin peptide (OXT) is not produced locally in taste tissue. **A**. RT-PCR was carried out on vallate papilla (t1), two samples of vallate and foliate taste buds (t2, t3), non-taste lingual epithelium (nt, negative control), and brain (br, positive control). Primers selective for OXT (top), PLCβ2 (middle) and β-actin (bottom) were tested in parallel for each sample. The expected PCR product and size in basepairs are indicated at right. **B,C**. Validating the anti-OXT antibody. Coronal sections of brain from heterozygous (*Oxt* +/−) or knockout (*Oxt* −/−) mice, fixed and processed in parallel for OXT-immunostaining (red). Sections through the hypothalamus reveal a cluster of OXT-positive neurons in the Paraventricular Nucleus (PVN), in *Oxt* +/− but not in *Oxt* −/− mouse. In **C**, the PVN is shown at higher magnification. **D**. Cryosections of vallate papillae from the same *Oxt* +/− and *Oxt* −/− mice as in B,C, immunostained with the same anti-OXT antibody (green). These sections were also immunostained with anti-Tyrosine Hydroxylase (TH, red) to reveal nerve fibers. Vertical arrows point at the apical pore of two taste buds in each panel. No specific immunofluorescence for OXT was detected in taste buds, adjacent epithelium, or nerve fibers. The faint green fluorescence in these images is identical in tissues from *Oxt* +/− and −/− mice and thus, cannot be attributed to OXT. Certain primary and secondary antibodies, even those well-validated in other tissues, do yield similar faint non-specific background staining in taste tissue. The “gold standard” test [58] in knockout tissue demonstrates that it is non-specific. Scale bars are 50 µm for B and 20 µm for C, D.

To evaluate the source of OXT that might influence taste buds *in vivo*, we performed RT-PCR and immunofluorescence to determine whether the peptide is synthesized or accumulates in taste tissue. With either method, we found no evidence for expression of OXT peptide in cells of taste buds or in adjacent epithelial and other tissues (Fig. 6A, D). We also did not detect OXT peptide in nerve fibers that approach or penetrate taste buds (Fig. 6D). Thus, we infer that OXT is delivered to taste buds via the circulation. This is similar to how leptin, another satiety peptide, influences taste sensitivity [36], [37], and how OXT reaches its other peripheral targets.

### Taste bud morphology in mice lacking OXTR

**Figure 7:**
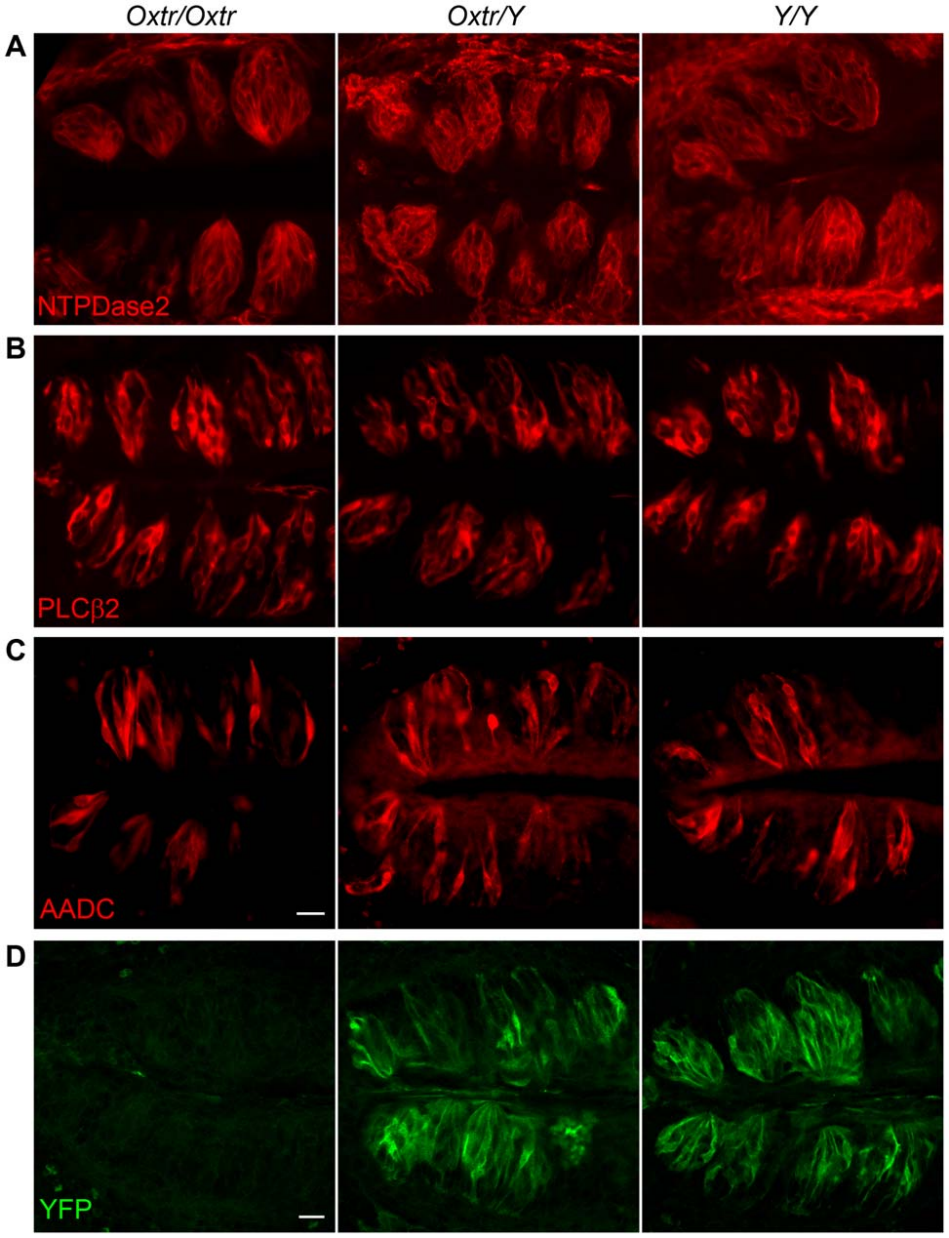
OXTR deficiency does not alter the morphology of taste buds. Vallate papillae were harvested from mice of the following genotypes: wildtype (*Oxtr/Oxtr*), heterozygous (*Oxtr/Y*), or homozygous for the OXTR-YFP knock-in allele (*Y/Y*). The *Y/Y* mice have no functional allele for OXTR, and are OXTR knockouts. We immunostained sections of vallate papillae for NTPDase2 (**A**), PLCβ2 (**B**), and aromatic amino acid decarboxylase (AADC) (**C**). NTPDase2 and PLCβ2 are markers for Glial-like and Receptor cells, respectively. AADC is a marker for Presynaptic cells [18], [29]. Sections from all three genotypes immunostained with anti-GFP (**D**) are shown for reference. We observed no difference in size, shape, or number of cells of each type across genotypes. Scale bars, 20 µm. Bar in C applies to A–C.

Oxytocin has been shown to induce differentiation of cardiomyocytes [38] and osteoblasts [39]. Given the expression of OXTR in peripheral cells (arrowheads in Fig. 2B–D; Fig. 3A), which may be immature or undifferentiated taste cells (see *Discussion*), we reasoned that a role for OXT could be to influence differentiation of taste cells. We therefore asked if there are obvious differences in the morphology of taste buds in mice deficient in OXTR. OXTR-YFP knock-in mice can possess either one copy of the *Oxtr* gene (i.e. *Oxtr*/*Y*) or zero copies (i.e. *Y/Y*). Thus, we immunostained taste tissue from *Oxtr/Oxtr* (i.e. wild-type), *Oxtr*/*Y* (heterozygous), and *Y/Y* (homozygous for *Oxtr*-YFP knock-in) mice. We then visualized Glial-like (Type I), Receptor (Type II), and Presynaptic (Type III) cells from each of these tissues (Fig. 7). We observed no significant differences in size, shape, or cell number for each of the cell types across the genotypes. The morphology was sufficiently similar that a detailed, quantitative analysis did not appear to be warranted. Lack of OXTR signaling does not appear to affect the structural development of taste buds or cells.

## Discussion

We show, using immunofluorescence and RT-PCR on taste buds and single-cells, that OXTR is found in a subset of Glial-like (Type I) cells of taste buds and also in some cells on the periphery of taste buds. Physiologically appropriate concentrations of bath-applied OXT evoked Ca^2+^ mobilization in Glial-like (Type I) taste cells and these responses were reversibly blocked by an OXTR-selective antagonist. Isolated taste cells that responded to OXT were neither Receptor (Type II) cells nor Presynaptic (Type III) cells, consistent with the molecular expression data. OXT is not produced locally in taste buds, surrounding cells, or nerves. It is likely to be delivered via the circulation under a variety of physiological conditions.

Of the identified taste cell types, only Type I cells express OXTR. Type I cells were originally defined ultrastructurally [20], [30]. More recently, these cells were proposed to be “Glial-like” on the basis of clearance mechanisms for two transmitters, ATP and glutamate [28], [40]. Although the ectonucleotidase, NTPDase2, is limited to Glial-like taste cells [28], it is not known to be a universal marker for *all* Glial-like cells. In OXTR-YFP taste buds, we found some YFP- labeled cells that were *not* NTPDase2-immunoreactive. Establishing whether such YFP-expressing cells are indeed Glial-like taste cells would require ultrastructural correlation of YFP with the characteristic “dark” electron-dense cytoplasm of Glial-like cells. On the other hand, PLCβ2 and Chromogranin A are expressed in all Receptor and Presynaptic cells, respectively [17], [18], [21], [29]. The lack of co-expression of these well-established markers with YFP in OXTR-YFP mice supports the conclusion that OXTR is not present in Receptor or Presynaptic cells.

Although Glial-like taste cells comprise approximately half the cells in the taste bud [30], very little is known about their functional roles. They appear to enwrap other cells within the taste bud, reminiscent of glia in the nervous system [20], [30]. We recently suggested that, in addition to transmitter clearance, they may also be involved in redistributing extracellular K^+^ [27], a role also typically subsumed by glia in nervous tissue. If the analogy with glia extends across a range of functional roles, then Glial-like cells might communicate with, and serve to modulate signaling by the excitable cells of taste buds, as occurs in the brain.

OXT is implicated in systemic salt balance by centrally regulating salt appetite and natriuresis [15]. OXT-KO mice consume more salt solution after overnight fluid deprivation than do wild-type mice [16]. The kidney is a known target of oxytocin, and rates of Na^+^ excretion are known to correlate with levels of plasma OXT [41]. Salt appetite is strongly influenced by central oxytocin while subcutaneously delivered OXT was reported not to affect saline intake in rats [42], [43]. Yet, OXT was reported to decrease transepithelial amiloride-sensitive Na currents (ASSCs), thought to represent salt taste transduction [44]. Interestingly, salt taste detection and the underlying ASSCs may be limited to a subset of Glial-like cells [45], [46]. Consistent with this, salt-sensing taste cells were reported to be distinct from those that sense sweet, bitter, and umami stimuli (i.e. Receptor cells), and sour stimuli (i.e. Presynaptic cells) [47]. The implication is that salt-sensing cells may represent a subset of Type I cells.

Apart from its role in regulating food and fluid intake generally, there is recent appreciation for the impact of OXT on the consumption of particular macronutrients. Over a span of several days, relative to wild-type controls, OXT-KO mice overconsume solutions rich in carbohydrates [8], [10] and artificial sweeteners [9]. In contrast, isocaloric lipid emulsions were consumed equally by mice of both genotypes [11]. The authors attributed the sweet preference phenotype to a role for OXT in central mechanisms of post-ingestive carbohydrate satiety. However, the presence of OXTR in taste buds was not previously documented. Our observations reported here raise the possibility that OXT, in addition to its actions on central circuits, may also act on taste buds to regulate sweet appetite.

The primary sensors for sweet taste are Receptor (Type II) taste cells whereas OXTR is found in Glial-like (Type I) taste cells. If OXT modulates the taste signal for palatable stimuli, this could reflect cell-to-cell communication between Glial-like cells and Receptor cells. Alternatively, OXT may influence the ability of Glial-like cells to alter the ionic environment [27] and secondarily influence Receptor or Presynaptic cells. This raises an interesting new parallel between glia in the central nervous system and in the taste bud. OXTR, co-expressed with glutamate-aspartate transporter (GLAST), was detected in glial cells in several areas of the brain [19]. GLAST is also expressed in most glial-like cells of taste buds [28], [40], which we show here are targets of oxytocin. In hypothalamic astrocytes, OXTR is instrumental in glial modulation of neuronal function [48]. We speculate that analogous mechanisms may be at work in the taste bud between Glial-like cells and the excitable taste-sensing Receptor or Presynaptic cells.

Our Ca-imaging studies show that OXT triggers Ca^2+^ responses in some taste bud cells. Curiously, the ATP-evoked Ca^2+^ responses in OXT-responsive taste cells were larger than those in OXT-nonresponsive taste cells (e.g. Figs. 4B–C; 5C). Taste buds removed from the epithelium (for functional imaging) do not fully retain their original morphology in the recording chamber. Nevertheless, OXT-responsive cells sometimes had an obvious peripheral location in the taste bud (e.g. Fig. 4A). Peripheral cells of fungiform taste buds have been reported to generate robust Ca^2+^ responses to ATP [33]. Although our physiological experiments were conducted in vallate, not fungiform, taste buds, our functional data and the peripheral location of YFP-expressing cells in fungiform taste buds (Figs. 2B–C) are consistent with the results of Yoshii and colleagues [33]. In combination, the observations raise the possibility that large ATP responses are a characteristic property of OXTR-expressing peripheral taste cells.

Parenthetically, in taste buds of OXTR-YFP mice, we found that some of the most intense YFP-labeled cells lacked NTPDase2 and were situated on the periphery of the taste bud (Fig. 2B, D, Figure S1). Peripheral cells have been interpreted as presumptive taste cells in the process of migrating from surrounding epithelium, integrating into the taste bud, and differentiating into taste cells [20]. In our studies, peripheral YFP-labeled cells frequently did not express known markers for mature taste cells (Fig. 2B–D, 3A arrowheads). Thus, we suggest that many OXTR-expressing cells may be partially differentiated or immature taste cells. In our immunohistochemical and RT-PCR analyses, we note that the incidence of YFP (Fig. 2, 3A,B) appears to be higher than the incidence of OXTR itself (Fig. 3D). This may reflect the stability of YFP protein. We suggest that OXTR (and YFP) may be transiently expressed during the differentiation of certain taste cells. The fluorescence of YFP may persist after OXTR itself is no longer detectable in more mature Glial-like Type I cells.

In closing, we can formulate two hypotheses from our discovery that a subset of Glial-like Type I cells expresses OXTR and is responsive to OXT. OXT may modulate the afferent sensory output of taste buds via Glial-like cells, or may influence the development and/or maintenance of taste buds, or both. Our observation that OXTR deficient mice have normal numbers of taste buds and Glial-like, Receptor, and Presynaptic cells does not support a prominent role for OXT in taste bud morphological development. However, there remains the possibility that OXT may contribute to the *functional* maturation of taste cells; this might not be reflected in their morphology. Future physiological studies will reveal whether OXT can modulate taste-evoked signals (within taste cells or gustatory nerves). Further, mice genetically modified to lack OXTR selectively in taste buds will be valuable to ask if OXT signaling is associated with alterations of signal processing within the taste bud.

The localization of OXTR in Glial-like taste cells rather than the primary taste sensors (Receptor and Presynaptic cells) raises the question of how OXT might alter taste-evoked responses. Both Receptor and Presynaptic cells use neurotransmitters to communicate in a paracrine fashion and modulate each other's responses to taste stimuli [22], [34], [49], [50]. There is no *a priori* reason to assume Glial-like Type I cells could not do likewise. Indeed, glia in the brain participate in modulating synaptic activity by releasing “gliotransmitters” [51], [52]. The Glial-like cells of taste buds have thus far remained poorly-explored compared to Receptor and Presynaptic cells, perhaps due to there being fewer clues as to what their active roles are. With this and our previous study [27], we aim to uncover new insights about these cells to reveal their functions in the taste bud.

## Materials and Methods

### Animals

We used five strains of transgenic mice: PLCβ2-GFP (GFP only in Receptor cells [53]; GAD1-GFP (GFP only in Presynaptic cells [23], [54]; PLCβ2-GFP x GAD1-GFP, a cross of the above two (GFP in both Receptor and Presynaptic taste cells [27]); OXTR-YFP knock-in (YFP replaces OXT coding sequence [19]); and OXT-knockout [55]. Procedures were approved by the Institutional Animal Care and Use Committees of University of Miami or Tohoku University.

### Physiological buffers

Regular Tyrode's solution consisted of (in mM): 145 NaCl, 5 KCl, 2 CaCl_2_, 1 MgCl_2_, 10 HEPES, 10 dextrose, 10 Na pyruvate, and 5 NaHCO_2_, pH 7.4 (315–325 mOsm). Ca^2+^/Mg^2+^ free Tyrode's solution was as above with 2 mM each EGTA and BAPTA replacing Ca^2+^ and Mg^2+^ salts. All reagents were from Sigma (St. Louis, MO).

### Taste bud and cell isolation

Mice were anesthetized with CO_2_ and cervically dislocated per NIH guidelines. Taste buds were harvested into fire-polished glass pipettes and were dissociated by gentle trituration to collect single taste cells [27]. Non-taste epithelium adjacent to the vallate papilla served as negative control in RT-PCR. Taste cells representing the three functional classes were collected as follows. GFP+ cells, collected from either PLCβ2-GFP or GAD1-GFP mice, were tentatively identified as Receptor or Presynaptic respectively. GFP-*lacking* cells from taste buds of PLCβ2-GFP x GAD1-GFP mice were tentatively identified as Glial-like. Cells were transferred to lysis buffer either singly or in pools of 20 cells of a single type. The cell-type identity of each cell or pool was confirmed by RT-PCR for marker mRNAs (PLCβ2 for Receptor cells, GAD1 or SNAP25 for Presynaptic cells, NTPDase2 for Type I cells).

### RT-PCR

Primers are listed in Table S1. RT-PCR (end-point and real-time) was carried out on 1–5 taste bud equivalents of cDNA or on single-cell cDNA as described [27]. For single-cell RT-PCR, we used 30% of the cDNA of each cell to confirm cell type (i.e. 10% cDNA in each of 3 reactions: NTPDase2, PLCβ2 and SNAP25). The remaining 70% of each single cell cDNA was used to test for expression of OXTR. In some instances, the cDNA of individual cells was subjected to T7 RNA polymerase-based linear amplification using the Message BOOSTER kit for qPCR (Epicentre, Madison, WI) [18].

### Immunostaining

Primary and secondary antibodies and validation of specificity are listed in Table S2. Mice were perfusion-fixed, tissue was cryosectioned and immunostained along with negative controls in parallel as described [27]. For OXT, perfusion was at pH 6.5 followed by pH 9.5 as recommended [56] and blocking was with 5% goat serum.

### Microscopy for Immunohistochemistry

Confocal micrographs (Figs. 2, 3, 6, 7D, S1, S2) were obtained on a Zeiss LSM510 Axiovert 200 M microscope, with ∼1–2 µm depth of field (optical slice). Widefield fluorescent images (Fig. 7A–C) were obtained on a Zeiss Microimaging Axioplan epifluorescence microscope. Brightness and contrast levels were adjusted in parallel for panels within each figure, using Adobe Photoshop CS4.

### Calcium Imaging

Taste buds and clusters of taste cells were isolated as above, deposited on coverslips and loaded with fura-2 AM as described previously [34]. Stimuli and drugs (OXT and L-371,257 from Tocris Bioscience, Ellisville, MO) were dissolved in Tyrode's solution, and presented via constant bath perfusion. The ratio of fluorescence intensities (F340/380) was calculated using Imaging Workbench v.5 software (INDEC Biosystems, Mountain View, CA). Responses were quantified as the difference between peak F340/380 less the average baseline for 10 s prior to stimulus application, as previously described [57]. Graphs were plotted and statistical analyses performed in Prism v.5 (Graphpad, La Jolla, CA).

## Supporting Information

Figure S1 YFP is seen in nerve fibers beneath the palatal epithelium of OXTR-YFP mice. Cryosections of palatal epithelium containing taste buds were immunostained for YFP (green) and PLCβ2 (red). As seen in Fig. 2, Receptor (PLCβ2+) cells are distinct from YFP+ cells in taste buds. Below the epithelium, fibrous structures resembling nerve fibers also show YFP fluorescence (arrowheads). Scale bar, 20 µm.
(1.15 MB TIF)

Figure S2 YFP-positive cells from OXTR-YFP mice have a distinctive morphology. Cryosections of vallate papilla were immunostained for YFP (green) and PLCβ2 (red) and counterstained with DAPI (blue) for nuclei. Receptor (PLCβ2+) cells have consistently smooth, ovoid cell bodies and nuclei with relatively distinct, thick processes. In contrast, OXTR-YFP cells tend to have irregularly shaped somata and nuclei (arrowhead). Thin, angular cytoplasmic processes of YFP-expressing cells penetrate the taste bud and extend some distance away from the cell body. Scale bar, 20 µm.
(1.64 MB TIF)

Table S1 Primers used for RT-PCR, derived from mouse (Mus musculus) cDNAs. * OXTR primers 1,2 were used for end-point and quantitative RT-PCR on all samples from lingual epithelium (Fig. 1), cell pools (Fig. 3C), and single cell samples from non-amplified RNA (Fig. 3D). OXTR primers 3, 4 amplify a sequence further towards the 3′ end of the cDNA, and were used on single-cell amplified RNA, where proximity to the 3′ end improves yield. †PLCβ2 primers 1,2 were used for end-point RT-PCR in Fig. 6A. PLCβ2 primers 3,4 were used in quantitative and end-point reactions (Fig. 1C, Fig. 3C).
(0.04 MB DOC)

Table S2 Primary and Secondary antibodies used, and their validation in earlier publications, or in Supporting material presented here. Each secondary antibody was validated through a no-primary negative control. 1.Trubey KR, Culpepper S, Maruyama Y, Kinnamon SC, Chaudhari N (2006) Tastants evoke cAMP signal in taste buds that is independent of calcium signaling. Am J Physiol Cell Physiol 291:C237-C244. 2. Rozengurt N et al. (2006) Colocalization of the alpha-subunit of gustducin with PYY and GLP-1 in L cells of human colon. Am J Physiol Gastrointest Liver Physiol 291:G792-G802. 3. Dvoryanchikov G, Tomchik SM, Chaudhari N (2007) Biogenic amine synthesis and uptake in rodent taste buds. J Comp Neurol 505:302-313. 4. Bartel DL, Sullivan SL, Lavoie EG, Sevigny J, Finger TE (2006) Nucleoside triphosphate diphosphohydrolase-2 is the ecto-ATPase of type I cells in taste buds. J Comp Neurol 497:1–12. 5. Tomchik SM, Berg S, Kim JW, Chaudhari N, Roper SD (2007) Breadth of tuning and taste coding in mammalian taste buds. J Neurosci 27:10840–10848.
(0.04 MB DOC)
