# Supplementary figures and images for: Oxytocin Signaling in Mouse Taste Buds
Source: PLoS One. 2010 Aug 5;5(8):e11980. doi: 10.1371/journal.pone.0011980 (PMC2916830; doi:10.1371/journal.pone.0011980)

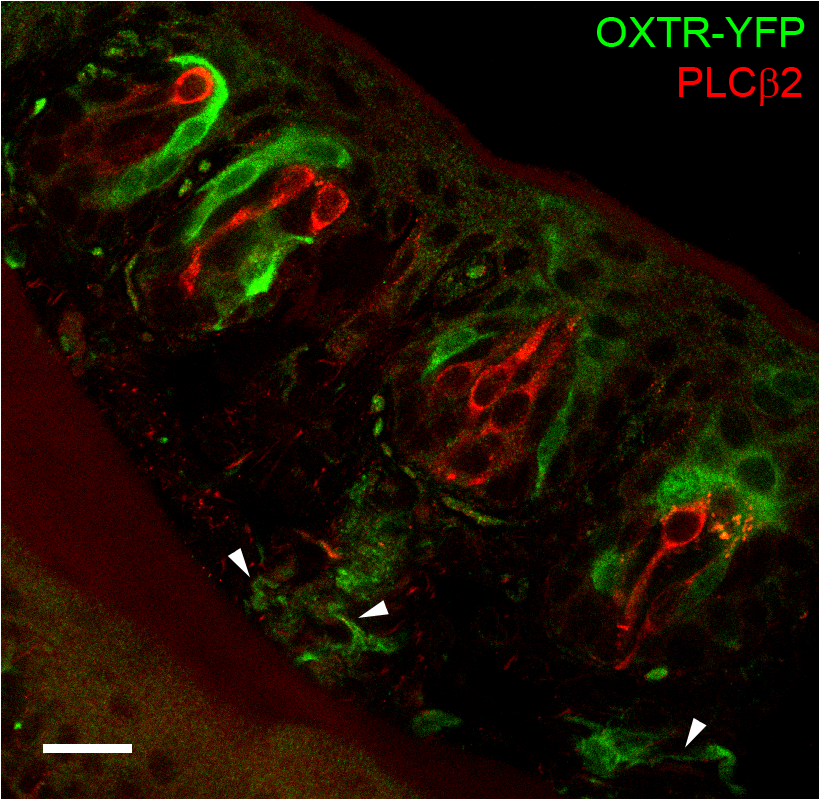

Supplement: Figure S1 — YFP is seen in nerve fibers beneath the palatal epithelium of OXTR-YFP mice. Cryosections of palatal epithelium containing taste buds were immunostained for YFP (green) and PLCβ2 (red). As seen in Fig. 2, Receptor (PLCβ2+) cells are distinct from YFP+ cells in taste buds. Below the epithelium, fibrous structures resembling nerve fibers also show YFP fluorescence (arrowheads). Scale bar, 20 µm. (1.15 MB TIF) [file pone.0011980.s001.tif]

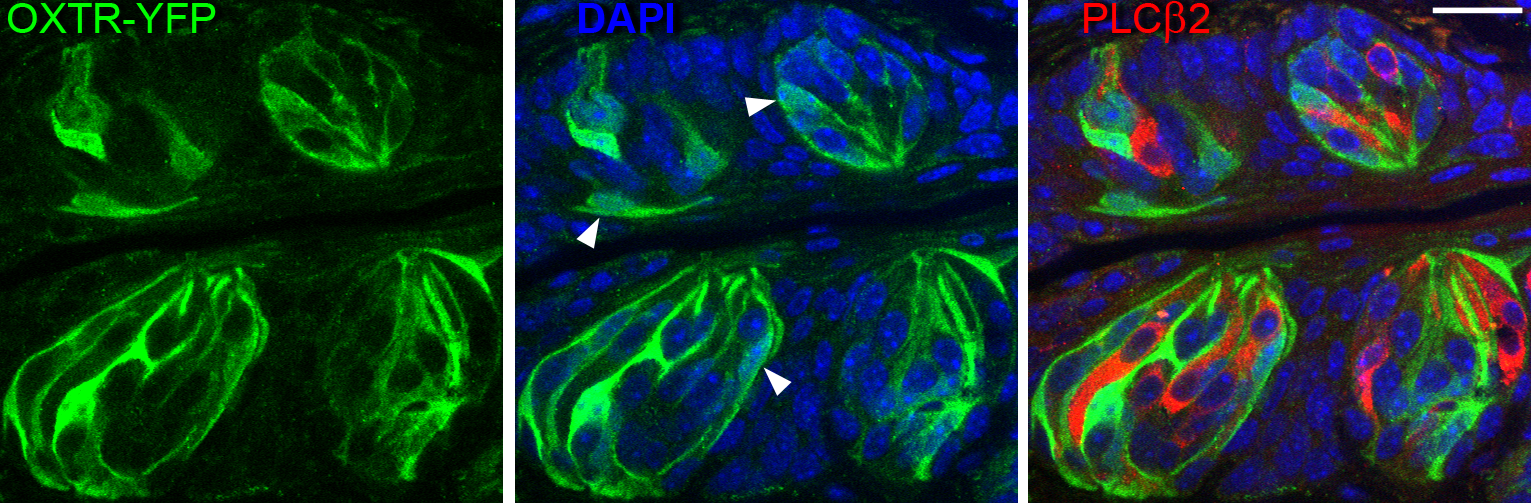

Supplement: Figure S2 — YFP-positive cells from OXTR-YFP mice have a distinctive morphology. Cryosections of vallate papilla were immunostained for YFP (green) and PLCβ2 (red) and counterstained with DAPI (blue) for nuclei. Receptor (PLCβ2+) cells have consistently smooth, ovoid cell bodies and nuclei with relatively distinct, thick processes. In contrast, OXTR-YFP cells tend to have irregularly shaped somata and nuclei (arrowhead). Thin, angular cytoplasmic processes of YFP-expressing cells penetrate the taste bud and extend some distance away from the cell body. Scale bar, 20 µm. (1.64 MB TIF) [file pone.0011980.s002.tif]
