# Supplementary material for: Oxytocin Signaling in Mouse Taste Buds
Source: PLoS One. 2010 Aug 5;5(8):e11980. doi: 10.1371/journal.pone.0011980 (PMC2916830; doi:10.1371/journal.pone.0011980)
Supplement: Table S1 — Primers used for RT-PCR, derived from mouse (Mus musculus) cDNAs. * OXTR primers 1,2 were used for end-point and quantitative RT-PCR on all samples from lingual epithelium (Fig. 1), cell pools (Fig. 3C), and single cell samples from non-amplified RNA (Fig. 3D). OXTR primers 3, 4 amplify a sequence further towards the 3′ end of the cDNA, and were used on single-cell amplified RNA, where proximity to the 3′ end improves yield. †PLCβ2 primers 1,2 were used for end-point RT-PCR in Fig. 6A. PLCβ2 primers 3,4 were used in quantitative and end-point reactions (Fig. 1C, Fig. 3C). (0.04 MB DOC) [file pone.0011980.s003.doc]

| Protein/*gene* | Accession # | Forward Primer (5’🡪3’) | Reverse Primer (5’🡪3’) | Product, bp | Anneal °C |
| --- | --- | --- | --- | --- | --- |
| β-actin / *Actb* | NM_007393 | caccctgtgctgctcacc | gcacgatttccctctcag | 328 | 58 |
| GAD1 / *Gad1* | NM_008077 | agatagccctgagcgacgag | atggccgatgattctggttc | 240 | 59 |
| NTPDase2 / *Entptd2* | NM_009849 | agctggaggatgccacagag | gagagcaacccaggagctga | 299 | 63 |
| OXT / *Oxt* | NM_011025 | cacctacagcggatctcagac | ctccgagaaggcagactcag | 407 | 61 |
| * OXTR / *Oxtr* | NM_001081147 | *Pr #1*  ttcttcgtgcagatgtggag | *Pr #2*  ccttcaggtaccgagcagag | 187 | 58 |
|  |  | *Pr #3*  tgtgtctccttttgggacaa | *Pr #4*  ggcatttcagaattggctgt | 233 | 58 |
| †PLCβ2 / *Plcb2* | NM_177568 | *Pr #1*  ctcgctttgggaagtttgc | *Pr #2*  gcattgactgtcatcgggt | 226 | 58 |
|  |  | *Pr #3* gagcaaatcgccaagatgat | *Pr #4*  ccttgtctgtggtgaccttg | 163 | 60 |
| SNAP25 / *Snap25* | NM_011428 | ggcaataatcaggatggagtag | agatttaaccacttcccagca | 310 | 58 |

**Table S1. Primers used for RT-PCR, derived from mouse (*Mus musculus*) cDNAs.**

*OXTR primers 1,2 were used for end-point and quantitative RT-PCR on all samples from lingual epithelium (Fig. 1), cell pools (Fig. 3C), and single cell samples from non-amplified RNA (Fig. 3D). OXTR primers 3, 4 amplify a sequence further towards the 3’ end of the cDNA, and were used on single-cell amplified RNA, where proximity to the 3’ end improves yield.

†PLCβ2 primers 1,2 were used for end-point RT-PCR in Fig. 6A. PLCβ2 primers 3,4 were used in quantitative and end-point reactions (Fig. 1C, Fig 3C).
