# Supplementary material for: Oxytocin Signaling in Mouse Taste Buds
Source: PLoS One. 2010 Aug 5;5(8):e11980. doi: 10.1371/journal.pone.0011980 (PMC2916830; doi:10.1371/journal.pone.0011980)
Supplement: Table S2 — Primary and Secondary antibodies used, and their validation in earlier publications, or in Supporting material presented here. Each secondary antibody was validated through a no-primary negative control. 1.Trubey KR, Culpepper S, Maruyama Y, Kinnamon SC, Chaudhari N (2006) Tastants evoke cAMP signal in taste buds that is independent of calcium signaling. Am J Physiol Cell Physiol 291:C237-C244. 2. Rozengurt N et al. (2006) Colocalization of the alpha-subunit of gustducin with PYY and GLP-1 in L cells of human colon. Am J Physiol Gastrointest Liver Physiol 291:G792-G802. 3. Dvoryanchikov G, Tomchik SM, Chaudhari N (2007) Biogenic amine synthesis and uptake in rodent taste buds. J Comp Neurol 505:302-313. 4. Bartel DL, Sullivan SL, Lavoie EG, Sevigny J, Finger TE (2006) Nucleoside triphosphate diphosphohydrolase-2 is the ecto-ATPase of type I cells in taste buds. J Comp Neurol 497:1–12. 5. Tomchik SM, Berg S, Kim JW, Chaudhari N, Roper SD (2007) Breadth of tuning and taste coding in mammalian taste buds. J Neurosci 27:10840–10848. (0.04 MB DOC) [file pone.0011980.s004.doc]

| **Primary Antibody** | **Source** | **Dilution** | **Specificity validated** |
| --- | --- | --- | --- |
| Rabbit anti-PLCβ2 | Santa Cruz Biotechnology  #sc-206 | 1:1000 | (1) |
| Goat anti-Chromogranin A | Santa Cruz Biotechnology  #sc-1488 | 1:200 | (2, 3) |
| Rabbit anti-NTPDase2 | J. Sévigny, Université Laval, Quebec, Canada. #mN2-36I6 | 1:1000 | (4) |
| Rabbit anti-AADC | Genetex, #GTX30448 | 1:1000 | (3) |
| Chicken anti-GFP | Aves Labs  #GFP-1020 | 1:2000 | (5) |
| Guinea pig anti-OXT | Chemicon/ Millipore  #AB15704 | 1:1000 | Fig.S2 |
| Rabbit anti-TH  (tyrosine hydroxylase) | Chemicon  #AB152 | 1:1000 | (3) |
|  |  |  |  |
| **Secondary Antibody** | **Source** | **Dilution** | **Fluor** |
| Goat anti-chicken IgG | Invitrogen A11039 | 1:2000 | Alexa-488 |
| Goat anti-rabbit IgG | Invitrogen A11012 | 1:1000 | Alexa-594 |
| Goat anti-guinea pig IgG | Invitrogen A-11073 | 1:1000 | Alexa 488 |
| Donkey anti-goat IgG | Invitrogen A11058 | 1:1000 | Alexa-594 |

**Table S2.** Primary and Secondary antibodies used, and their validation in earlier publications, or in Supporting material presented here. Each secondary antibody was validated through a no-primary negative control.

1. Trubey KR, Culpepper S, Maruyama Y, Kinnamon SC, Chaudhari N (2006) Tastants evoke cAMP signal in taste buds that is independent of calcium signaling. Am J Physiol Cell Physiol 291:C237-C244.

2. Rozengurt N et al. (2006) Colocalization of the alpha-subunit of gustducin with PYY and GLP-1 in L cells of human colon. Am J Physiol Gastrointest Liver Physiol 291:G792-G802.

3. Dvoryanchikov G, Tomchik SM, Chaudhari N (2007) Biogenic amine synthesis and uptake in rodent taste buds. J Comp Neurol 505:302-313.

4. Bartel DL, Sullivan SL, Lavoie EG, Sevigny J, Finger TE (2006) Nucleoside triphosphate diphosphohydrolase-2 is the ecto-ATPase of type I cells in taste buds. J Comp Neurol 497:1-12.

5. Tomchik SM, Berg S, Kim JW, Chaudhari N, Roper SD (2007) Breadth of tuning and taste coding in mammalian taste buds. J Neurosci 27:10840-10848.
